# Conservative Management of Unusual Keratinising Squamous Metaplasia of the Bladder in a 28-Year-Old Female and Overview of the Literature

**DOI:** 10.1155/2012/940269

**Published:** 2012-07-08

**Authors:** Fernando Vázquez Alonso, Raquel Berrio Campos, Ignacio Puche Sanz, Manuel Segura Sánchez, Jose Miguel Molina Hernandez, Jose Francisco Flores Martin, Jose Manuel Cózar Olmo

**Affiliations:** Servicio de Urología, Hospital Universitario Virgen de las Nieves, Avenida de las Fuerzas Armadas, No. 2, 18014 Granada, Spain

## Abstract

Keratinizing squamous metaplasia of the bladder is rare and is usually associated with urinary tract infections and chronic irritation. It is considered a precancerous condition of squamous cell carcinoma, especially when more than 50% of the bladder surface is affected. Medical treatment cannot eradicate this lesion. When it is limited to a small area of the bladder, transurethral resection is possible. Annual cystoscopy with multiple biopsies as well as annual upper tract imaging is proposed in the follow up of these patients. We present a preliminary 2-year followup report of a keratinizing squamous metaplasia of the bladder in a 28-year-old female patient with no previous risk factors.

## 1. Introduction

Keratinizing squamous metaplasia (KSM) of the bladder is a rare entity which is mostly found in males. Risk of invasive carcinoma, retractile bladder, or obstructive uropathy appears when the lesion persists over time [1]. Treatment and subsequent follow-up management are still controversial. 

## 2. Case Presentation

**Figure 1:**
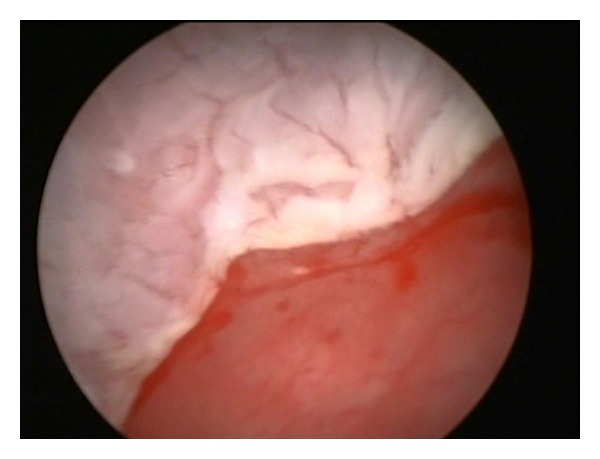
The cystoscopy revealed a whitish thin plaque clearly differentiated from the normal mucosa of the bladder trigone.

**Figure 2:**
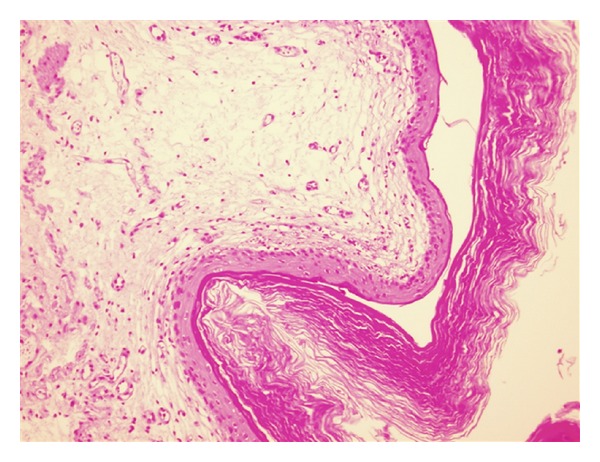
Squamous metaplasia of the bladder covered by a thick keratin layer.

A 28-year-old female was referred to our practice complaining with recurrent urinary symptoms such as dysuria, burning, pollakiuria, and urinary urgency. Neither personal nor familiar medical history of interest was reported. Periodical urocultures resulted negative. Yeasts and Koch's bacillus were also excluded. Intravenous urography (IVU) revealed a left double excretory system, without any other relevant findings. A CT scan showed an unspecific bladder wall thickening. A subsequent cystoscopy revealed a whitish thin plaque covering the whole bladder surface with the exception of the trigone area. Underneath this whitish plaque, the mucosa appeared erythematous and bled easily during the examination (Figure 1). Random biopsies reported a keratinizing squamous metaplasia of the bladder (Figure 2). Due to the age and mild symptoms of the patient, we agreed on a conservative management and proposed annual cystoscopy with random biopsies combined with annual upper urinary tract imaging (CT scan or IVU). After a two-year followup, the patient is felling generally well with occasional urinary symptoms but no evidence of disease progression.

## 3. Discussion

Squamous metaplasia of the bladder is defined as the transformation of the normal urothelium into stratified squamous epithelium and can be either keratinizing or not. Nonkeratinizing squamous metaplasia (N-KSM) occurs normally in the trigone of the bladder. It is also known as vaginal metaplasia and is mostly found in females. It is not usually associated with chronic irritation and has a characteristic lack of cellular atypia. N-KSM is considered an anatomic variation, caused by hormonal influx, and do not have clinical significance [1–3]. 

On the other hand, keratinizing squamous metaplasia (KSM) is considered a preneoplastic lesion. Chronic irritation is known to play an important role in the etiopathogenesis of KSM, and recurrent urinary infections seem to be the most important risk factor [1, 4]. The use of long-term catheterization, urinary lithiasis, chronic urinary tract obstruction, urinary fistulae, bladder exstrophy, neurogenic bladder, previous bladder surgery, pelvic radiotherapy, parasite colonization, and vitamin A deficiency are also associated with this disease [1, 2, 5, 6]. Only in a few cases no previous irritation or infectious factors are identified. Our case is one of those.

KSM usually presents with haematuria, dysuria, and urgency. Urinary obstruction, flank pain, and urinary elimination of a white flaky are less frequent. The characteristic cystoscopic finding is a whitish plaque, which can appear either localized or diffuse in the bladder. The mucosa underneath these plaques can bleed easily during the examination. Keratinization process can affect any area of the bladder, although it characteristically respects the ureteral orifices. However, endoscopic findings are not pathognomonic, and microscope examination is needed to exclude other diagnoses such as nonkeratinizing squamous metaplasia, fungal cystitis, malacoplakia, or amyloidosis [1, 7].

Natural history, prognosis, and therapeutic implications of squamous metaplasia of the bladder are currently under discussion. Squamous cell carcinoma is the most commonly associated neoplasia, and about 21% to 42% risk to develop a bladder cancer has been estimated [1]. Therefore, KSM is acknowledged to be a preneoplastic condition by most of authors, especially when the lesion persists over time and affects more than 50% of the bladder surface. Furthermore, in those cases in which KSM is associated with cellular dysplasia, the gradation of the lesion reaches a preinvasive status. Whenever metaplasia is found in transitional cell carcinomas and undifferentiated carcinomas, it implies a worse prognosis [8, 9]. Metaplasia has also been associated with the development of a retracted bladder, especially in long-time persistent lesions [1].

An increase in epithelial growth factor receptor (EGFR) activity was reported in those squamous carcinomas associated with previous squamous metaplasia of the bladder. Therefore, the EGFR has been proposed as a screening marker to identify squamous lesions, as well as a possible therapeutic target in the future [3].

Although spontaneous recovery of the bladder mucosa from the squamous metaplasia is possible, transurethral resection of the bladder in located lesions is a usual and feasible approach. Medical treatments such as oral vitamin A and silver nitrate, or acetic acid bladder instillations, have been tried with no promising results. Antibiotics could induce a symptomatic remission but cause no objective modifications in squamous metaplasia [1]. 

Bladder squamous cell carcinoma is frequently diagnosed in advanced stages, appearing normally to be unresectable by transurethral approach [10]. In those cases, in which radical cystectomy is undertaken, the prognosis of squamous cell carcinoma may be similar to the prognosis of conventional urothelial carcinoma with a cancer-specific survival of 57% at 5 years. 

In order to make an early diagnosis of squamous carcinoma, an annual monitoring by carrying out cystoscopies with multiple bladder biopsies has been suggested, as well as upper urinary system imaging, to detect possible complications derived from bladder keratinization. Radical cystectomy might be offered in cases of retractable bladder, disease progression, or even just in patients with a life expectancy greater than 10 years [1]. In our case, however, we chose a conservative approach with annual monitoring, due to the psychological and quality-of-life consequences that a prophylactic radical cystectomy could have caused in such a young patient. 

In conclusion, KSM of the bladder is considered a preneoplastic lesion which needs for special surveillance, due both to its potential malignancy and also to the possible complications caused by the progressive keratinization of the bladder. Medical treatment does not eliminate the lesion, and transurethral resection is the most recommended option when it is limited to an area of the bladder. If conservative approach is chosen, we propose a thorough monitoring by using an annual cystoscopy with random bladder biopsies and annual upper urinary system imaging. We think that prophylactic radical cystectomy should only be considered in selected, well-informed patients.

## References

[1] Khan MS, Thornhill JA, Gaffney E, Loftus B, Butler MR (2002). Keratinising squamous metaplasia of the bladder: natural history and rationalization of management based on review of 54 years experience. *European Urology*.

[2] Tramoyeres-Galvañ A, Canovas Ivorra JA, Sánchez-Ballester F (2005). Case report of bladder squamous metaplasia. Literature review. *Archivos Espanoles de Urologia*.

[3] Guo CC, Fine SW, Epstein JI (2006). Noninvasive squamous lesions in the urinary bladder: a clinicopathologic analysis of 29 cases. *American Journal of Surgical Pathology*.

[4] Özbey I, Aksoy Y, Polat O, Biçgi O, Demirel A (1999). Squamous metaplasia of the bladder: findings in 14 patients and review of the literature. *International Urology and Nephrology*.

[5] Castillo CM, Ha CY, Gater DR, Grob BM, Klausner AP (2007). Prophylactic radical cystectomy for the management of keratinizing squamous metaplasia of the bladder in a man with tetraplegia. *Journal of Spinal Cord Medicine*.

[6] Liang FX, Bosland MC, Huang H (2005). Cellular basis of urothelial squamous metaplasia: roles of lineage heterogeneity and cell replacement. *Journal of Cell Biology*.

[7] Ahmad I, Barnetson RJ, Krishna NS (2008). Keratinizing squamous metaplasia of the bladder: a review. *Urologia Internationalis*.

[8] Benson RC, Swanson SK, Farrow GM (1984). Relationship of leukoplakia to urothelial malignancy. *Journal of Urology*.

[9] Vecchioli Scaldazza C, Morosetti D (1994). Prognostic significance of epidermoid metaplasia in urothelial carcinoma of the bladder. *Archivos Espanoles de Urologia*.

[10] Lagwinski N, Thomas A, Stephenson AJ (2007). Squamous cell carcinoma of the bladder: a clinicopathologic analysis of 45 cases. *American Journal of Surgical Pathology*.

